# HEALTH PROFESSIONAL STUDENT-LED SOCIAL PHONE CALLS TO ADDRESS LONELINESS IN AGING-IN-PLACE ADULTS AFTER STROKE

**DOI:** 10.1093/geroni/igad104.3380

**Published:** 2023-12-21

**Authors:** Jason Burnett, Sean Savitz, Jordan Broussard, Jennifer Beauchamp

**Affiliations:** University of Texas Health Science Center at Houston, Houston, Texas, United States; University of Texas Health Science Center at Houston, Houston, Texas, United States; University of Texas Health Science Center at Houston, Houston, Texas, United States; University of Texas Health Science Center at Houston, Houston, Texas, United States

## Abstract

Social isolation and loneliness (SIL) increases the risk of stroke and recurrence by 32% and 40%, respectively. The functional and cognitive deficits that often accompany stroke can impair social engagement behaviors and increase SIL. Intergenerational programs have proven effective for addressing SIL through social engagement behavior change in older adults, but the feasibility of these programs is unspecified for aging-in-place adults after stroke. We paired 14 community-living adults after stroke with 14 health professional students (HPS) for 6-weekly unstructured social phone calls. Baseline and follow-up data measuring SIL and other psychosocial metrics were conducted. HPSs journaled following each call. The 13 (93%) participants completing all calls were an average of 57 years old and white non-Hispanic (77%). The calls lasted approximately 49 minutes. Average baseline scores for loneliness and social isolation were 48 and 22, respectively. The average score for loneliness decreased 4 points at week six. The average score for social isolation was unchanged. Average baseline scores for symptoms of depression and anxiety were 20 and 8, respectively, and scores decreased by 1 point at week six. Participants disclosed physical and emotional challenges, previous valued employment, and enjoyment from the social engagement. HPSs learned about the struggles of aging-in-place after stroke and valuing compassionate care and social engagement. An intergenerational social phone call program provided a feasible, acceptable, and encouraging approach for addressing loneliness in community-living adults after stroke and has encouraging effects on mental health. A randomized controlled trial is needed to provide definitive data on intervention effects.

